# Supplementary material for: Photoacoustic Tomography with Temporal Encoding Reconstruction (PATTERN) for cross-modal individual analysis of the whole brain
Source: Nat Commun. 2024 May 18;15:4228. doi: 10.1038/s41467-024-48393-z (PMC11102525; doi:10.1038/s41467-024-48393-z)
Supplement: Supplementary file 1 — Supplementary Information [file 41467_2024_48393_MOESM1_ESM.pdf]

Supplementary Information

Photoacoustic Tomography with Temporal Encoding Reconstruction (PATTERN) for Cross-Modal Individual Analysis of the Whole Brain

Authors: Yuwen Chen[1], Haoyu Yang[1], Yan Luo[1], Yijun Niu[1], Muzhou Yu, Shanjun Deng, Xuanhao Wang, Handi Deng, Haichao Chen, Lixia Gao, Xinjian Li, Pingyong Xu, Fudong Xue, Jing Miao, Song-Hai Shi, Yi Zhong, Cheng Ma*, Bo Lei*

[1] These authors contributed equally.
*Correspondence: **Bo Lei**: b.lei.2022@hotmail.com; **Cheng Ma**: cheng_ma@tsinghua.edu.cn

This file includes:

Supplementary Notes 1–2

Supplementary Figures 1–23

Supplementary Table 1

Supplementary Method 1

Supplementary Notes

Supplementary Note 1:

The adoption of half-ring array ultrasound transducers has alleviated the limited-view problem within the imaging plane, i.e., within the first two dimensions, all spatial frequency components are captured by the 180° coverage of the ultrasound array. Introducing translation along the elevational direction added a third dimension; however, the small numerical aperture corresponding to the elevational direction results in significant limited-view artifacts. To address this problem, the rotational scanning employs a multi-view strategy to synthetically create a large acceptance angle in the third dimension, effectively achieving nearly omnidirectional sampling in the spatial-frequency domain and minimizing the impact of the limited-view problem.

Supplementary Note 2:

The photoswitching-based technique is another promising alternative for fluorescent tagging. The rapid switching rate and potential for multiple cycles in the time domain translate into a narrower modulation bandwidth in the time-frequency domain. The narrow bandwidth facilitates the suppression of noise, thereby enhancing both sensitivity and specificity in distinguishing between fluctuating objects compared to the methods presented in the current work. Nevertheless, achieving this reversible capability relies on changes in the protein conformation of specific types of fluorescent tags. On the contrary, the bleaching-based method, employing routine labeling strategies, can be applied to nearly all currently available fluorescent proteins.

Supplementary Figures

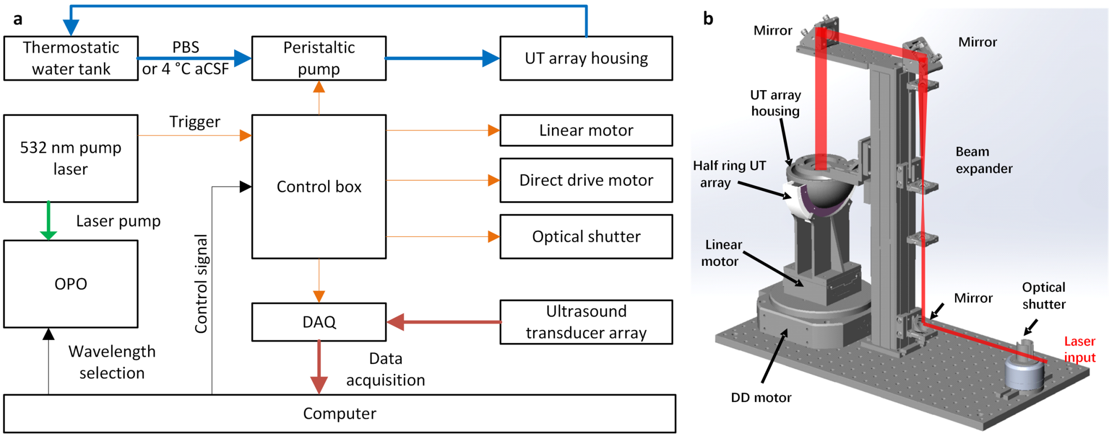

Supplementary Fig. 1 | Diagram and CAD model of PATTERN

a, Diagram of PATTERN. The optical shutter is used to control the expose to the sample and avoid bleaching curve distortion caused by extra laser pulse. For red fluorescent protein like mScarlet, the 532 nm pump laser was used as the excitation laser source **b**, CAD model of PATTERN. The excitation laser was expanded to match the scale of different brains.

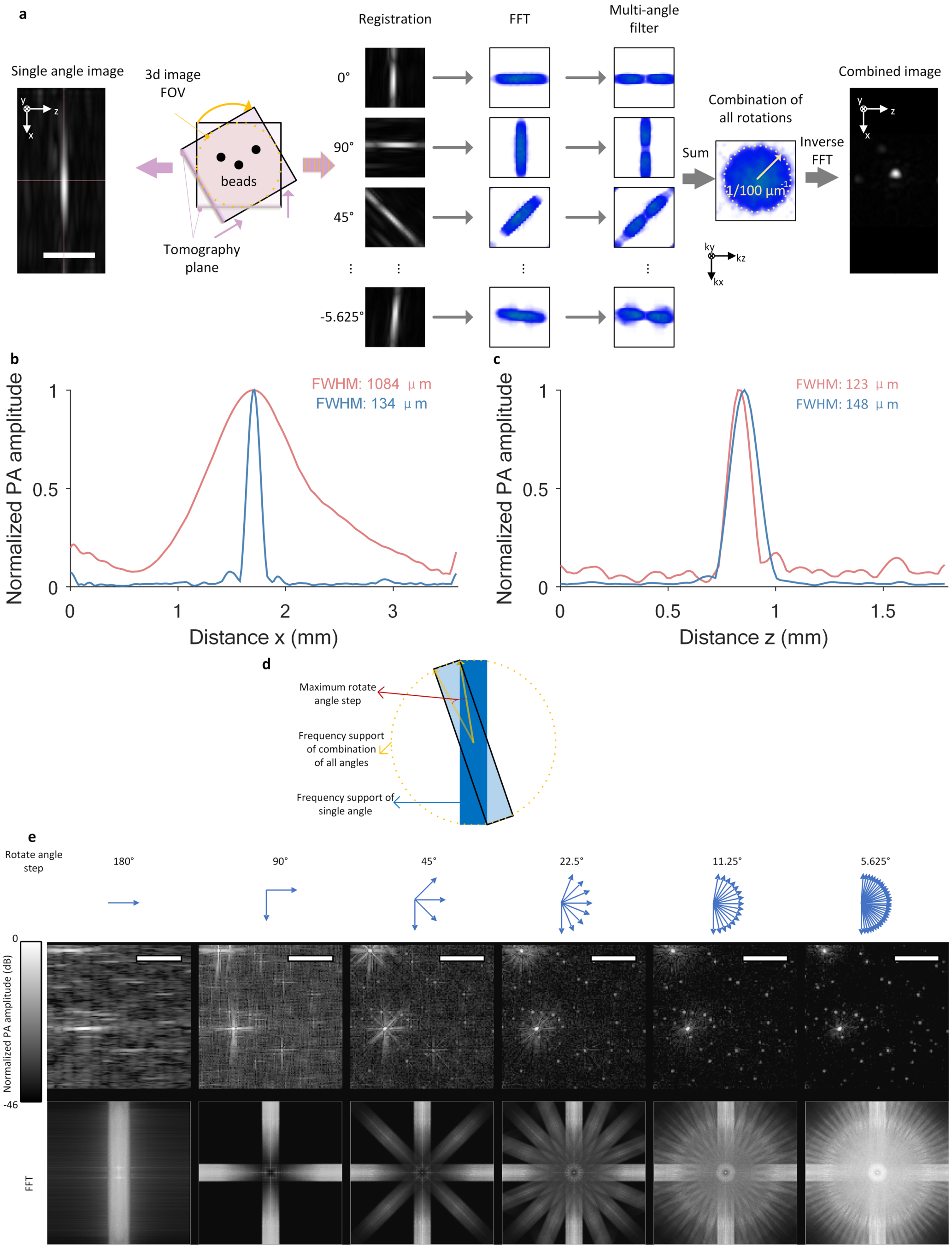

Supplementary Fig. 2 | Multiangle fusion process for PATTERN

a, Single angle reconstruction (left) versus multiangle reconstruction of beads. Scale bars, 1 mm **b**, Profiles of the vertical line related to (a). **c**, Profiles of the horizontal line related to (a). Red line: single angle, blue line: multiangle. **d**, Sketch of maximum allowed rotation step. **e**, Rotate directions (first row) with corresponding reconstructed image (second row) and their FFT (third row), illustrating the progressive improvement for both elevational resolution and SNR via decreasing rotation step (increasing rotation number). Scale bars, 3 mm

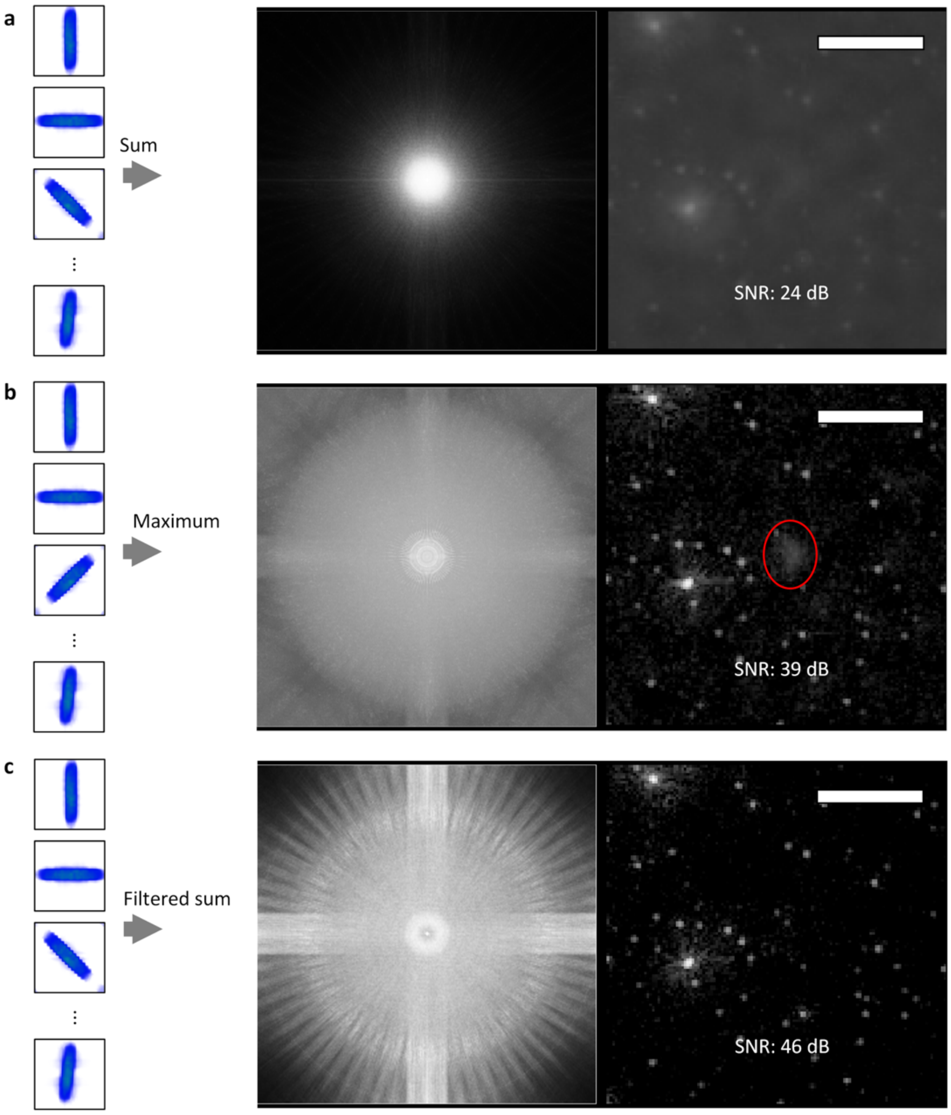

Supplementary Fig. 3 | SNR improvement via multiangle fusion for PATTERN

a, Directly sum all of the single angle 3D image **b**, Perform FFT to each 3D image and take the maximum value of every spatial frequency in k-space, then do inverse FFT to get the volume. **c**, Apply a multi-angle filter to each 3D image and then sum. The direct sum method in (a) over augments low spatial frequency thus blur the image. The method in (b) is relatively a theoretically optimal solution, but the slight misalignment of 3D images of different angles rise strong phase noise in k-space, resulting in some artefacts (red circle in (b)). Scale bars, 3mm

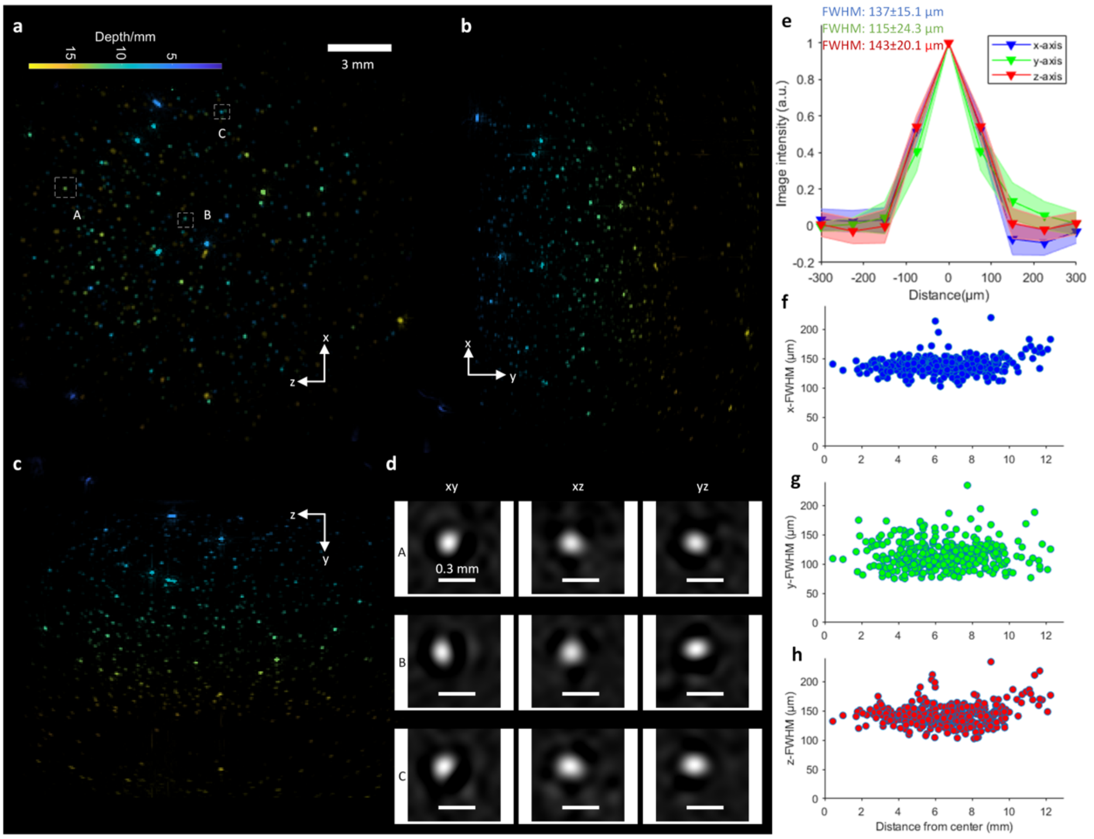

Supplementary Fig. 4 | Point spread function measurements

a-c, MIP images of red latex beads of 20 μm diameter embedded in 1.8% v/v agarose. **d**, Zoom in of slice view of beads corresponding to the dashed box in (a). The PSFs located different place inside of the FOV were slightly different. **e**, Averaged profiles of each bead along x, y and z direction. The shadows show the standard deviation of the profiles. The centers of qualified beads were firstly identified in the volume with a total number of N = 345 beads. And each bead was fit to a Gaussian distribution to quantify the FWHMs. **f-h**, FWHMs plotted versus distance from the center of the image (on the rotate axis of PATTERN). The results illustrate that the resolution of PATTERN keeps smooth inside the whole FOV of ~25 mm.

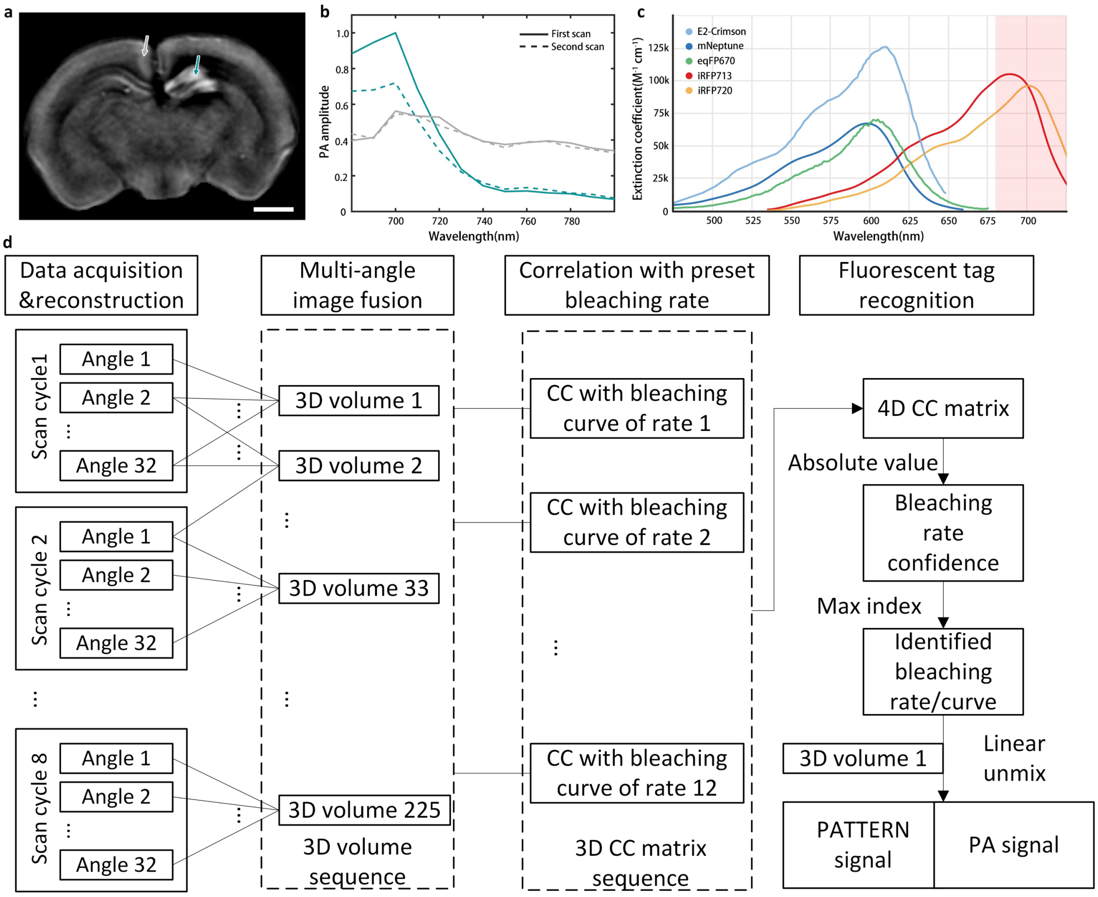

Supplementary Fig. 5 | Temporal encoding and unmixing method for PATTERN

a, A typical coronal slice of mouse brain with fluorescent protein. DarkCyan arrow indicates a pixel containing both intrinsic PA contrast and fluorescent protein contrast. Gray arrow indicates a pixel of only PA background. scale bar, 2 mm **b**, Spectrum of pixels related to the arrows in (a), solid line shows the first spectral sweeping results and dashed line shows the second, the bleaching of fluorescent protein was obvious. **c**, Excitation spectrum of commonly used fluorescent tags. Pink shadow, the sweeping window of our laser. **d**, Unmixing process for PATTERN. CC, correlation coefficient. The 3D volumes were interpolated from adjacent scan cycles thus providing more temporal sampling point. Instead of do curve fitting for every spatial point, we do correlation with several preset bleaching curve and use linear unmixing method to calculate the fitting parameters. Though it is still a point-by-point algorithm, the correlation procedure can be accelerated with GPU by more than 100 times.

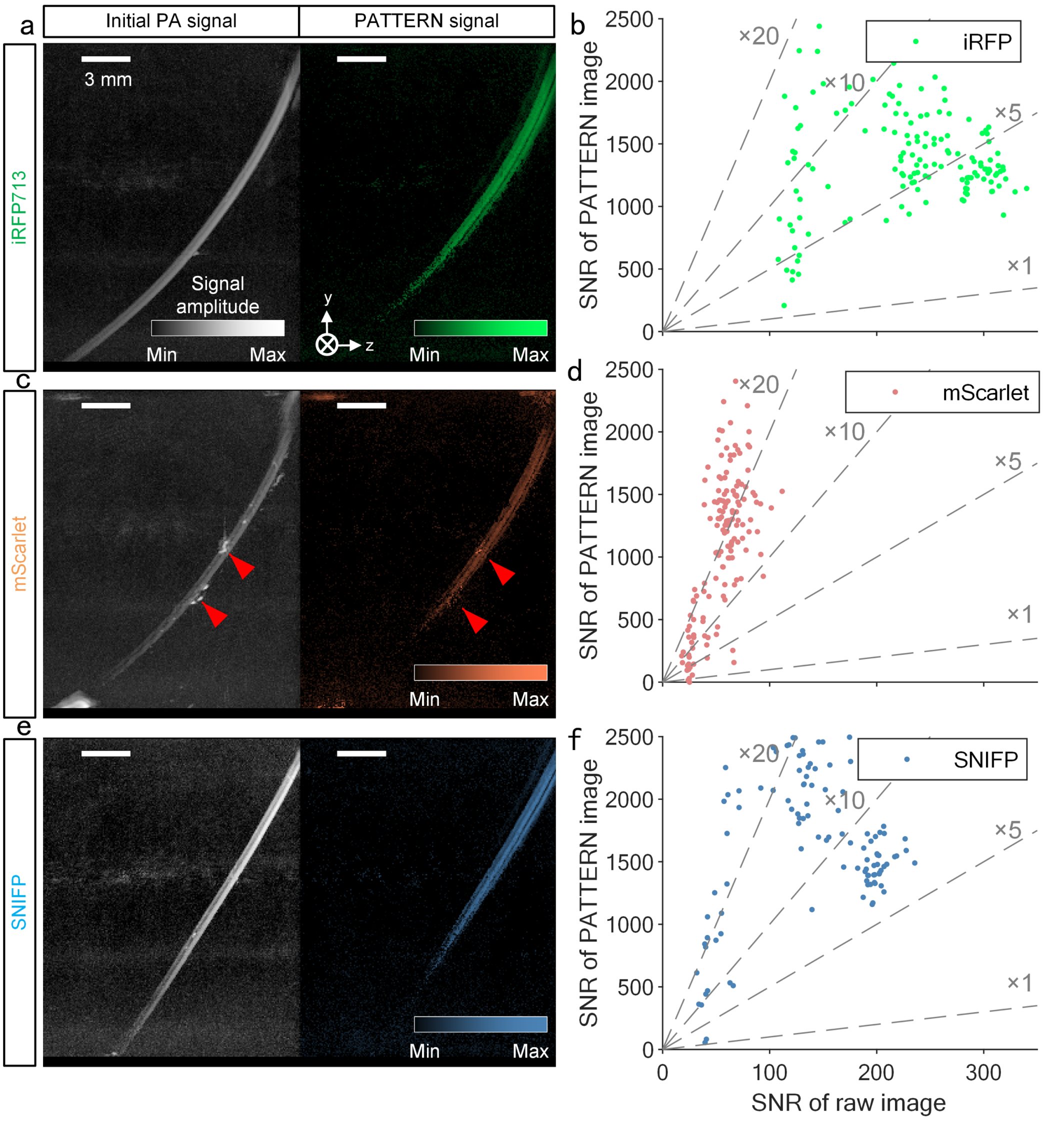

Supplementary Fig. 6 | Sensitivity enhancement of different fluorescent proteins.

a, Imaging results of iRFP713. **b**, SNR enhancement by PATTERN for iRFP713. **c**, Imaging results of mScarlet. **d**, SNR enhancement by PATTERN for mScarlet. **e**, Imaging results of SNIFP. **f**, SNR enhancement by PATTERN for SNIFP.

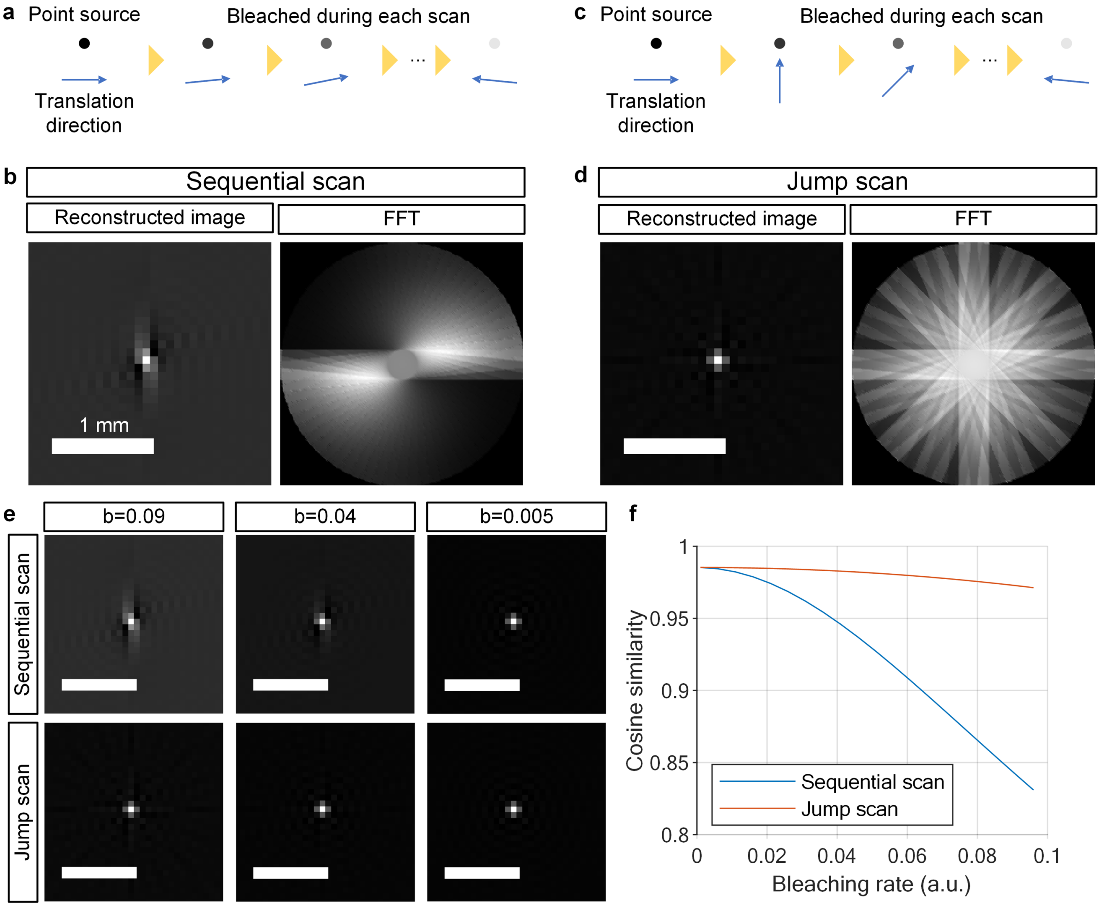

Supplementary Fig. 7 | Optimization of rotation order for image quality

a, Diagram of sequential scan of a point source which is exponentially bleached during the scan cycle. **b**, Reconstruction results from the scan and related FFT graph. **c**, **d**, The same as (a) and (b) but with jump scan. **e**, Reconstruction results with different bleaching rates. **f**, Cosine similarity between the point source image and the reconstructed image using sequential and jump scan.

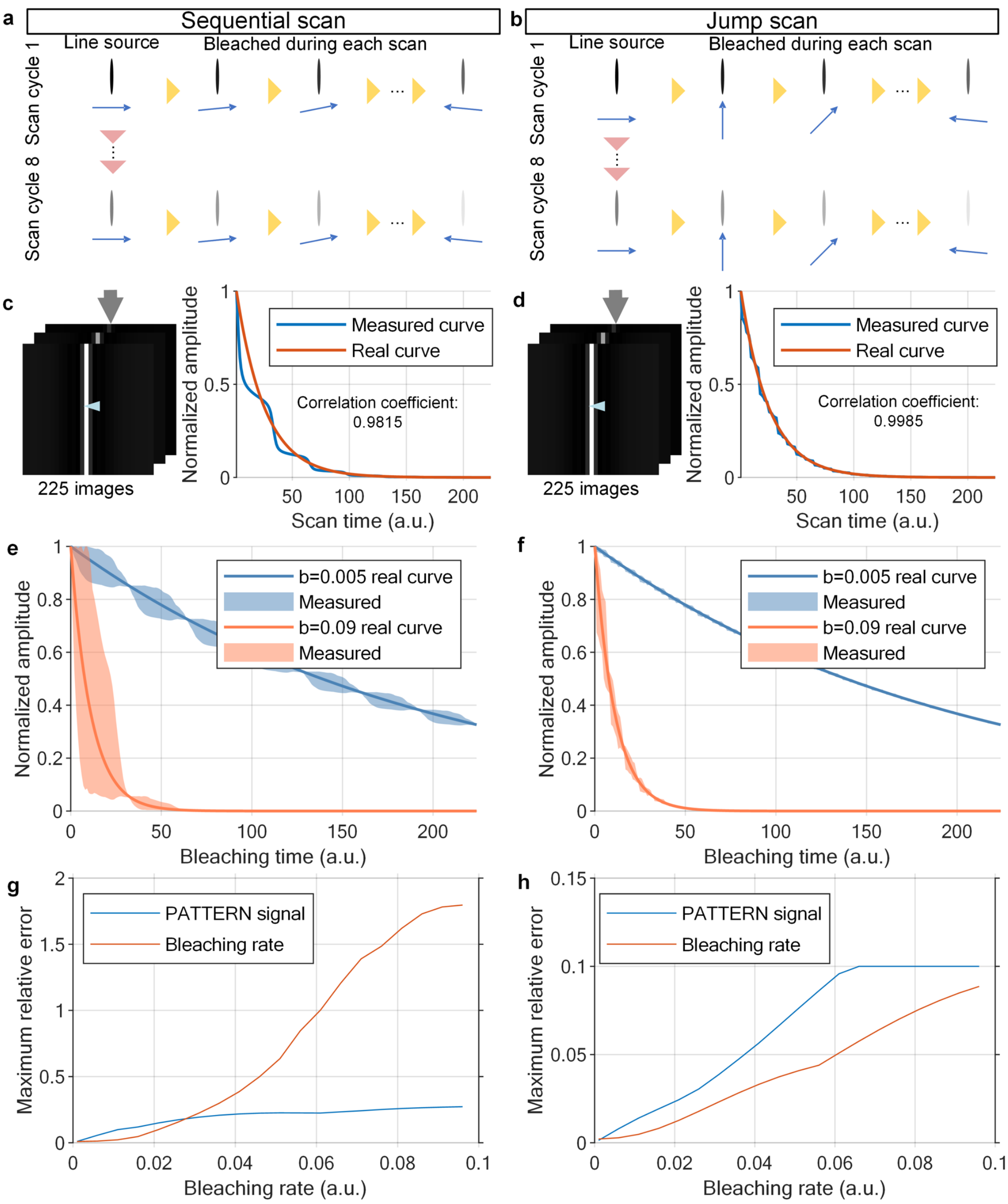

Supplementary Fig. 8 | Optimization of rotation order for quantification

a, Diagram of sequential scan of a line source which is exponentially bleached during the 8 scan cycles. **b**, The same as (a) but with jump scan. **c**, The bleaching curve calculated from the reconstruction results. **d**, The same as (c) but with jump scan. **e**, **f**, Measured distortion of bleaching curve induced by feature's directionality: (e) sequential scan, (f) jump scan. **g**, **h**, Maximum relative error of PATTERN signal amplitude and bleaching rate, induced by feature's directionality: (g) sequential scan, (h) jump scan.

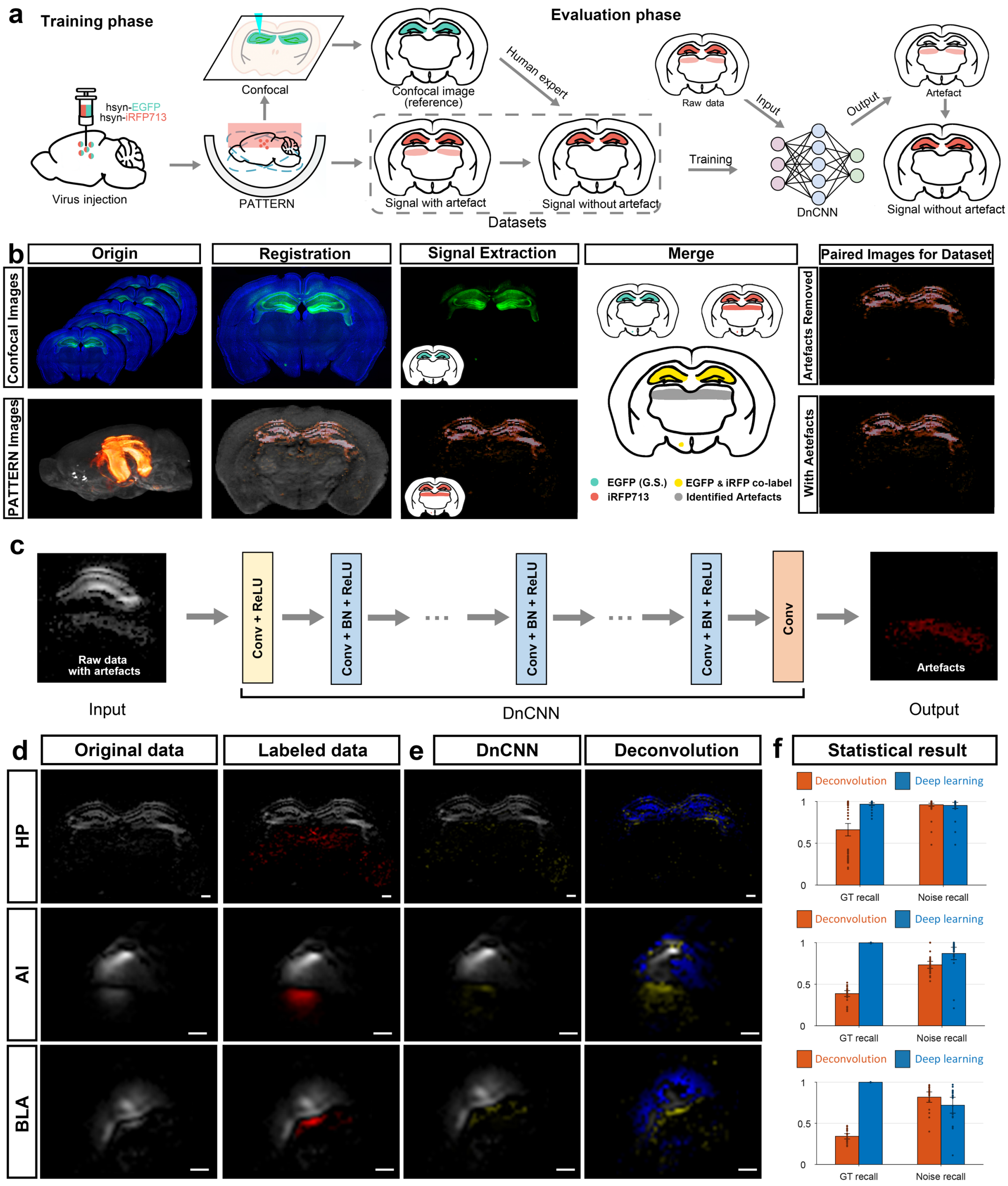

Supplementary Fig. 9 | Working flowchart and performance of DnCNN on artefact elimination.

a, Illustration of the workflow of DnCNN. Datasets are constructed in the training phase based on confocal microscopy as the reference. Network after training are used in the evaluation phase to remove the artefacts. **b**, Pipeline of artefact identification. Images with artefacts and the artefacts-removed ones were collected as datasets. **c**, The architecture of the proposed DnCNN network. The artefacts will be extracted from the original input. **d**, Representative original and labeled PATTERN data in HP, AI and BLA. Scale bars, 500 μm **e**, Processing results of images in figure c after DnCNN or deconvolution methods. Gray points represent the PAFL signal, red represent the artefacts, yellow represents

artefacts that were not removed correctly and blue represents real signals that were removed incorrectly. **f**, Statistic results of the processing results. GT recall represents the true signal retention rate; Noise recall represents the artefact removal rate. n=77,29,23 for HP,AI,BLA, biologically independent image slices. Error bar length: 2 * standard error of the mean (SEM). Source data are provided as a Source Data file.

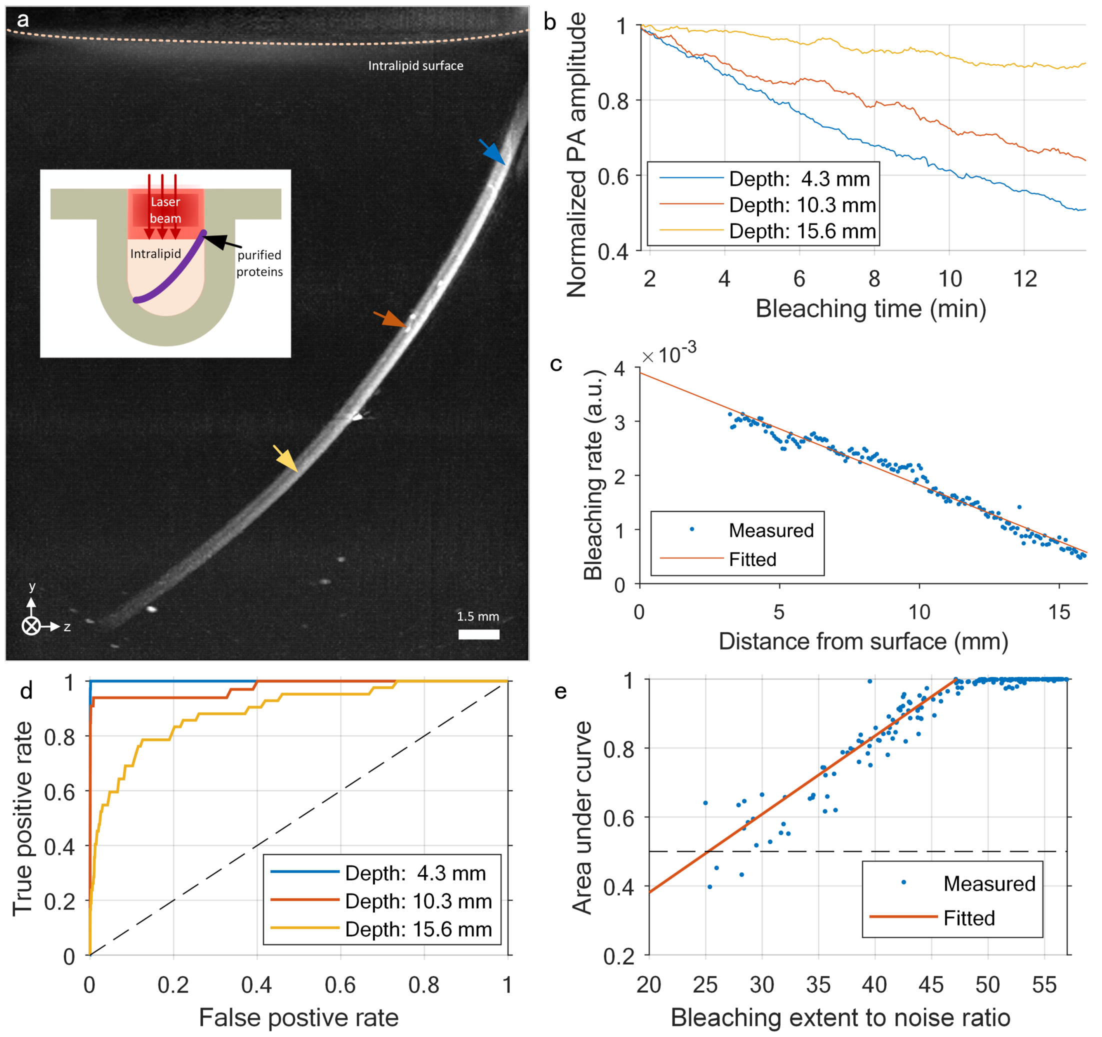

Supplementary Fig. 10 | Optimization of bleaching rates.

a, Imaging of a PTFE tube fulfilled with purified iRFP713, the inset shows the experimental schematic. **b**, Bleaching curves corresponding to different depths are indicated by the arrows in (a). **c**, Bleaching rate plotted against depth. d, ROC curve of three depths indicated by the arrows in (a). **e**, AUC of voxels with different bleaching extent.

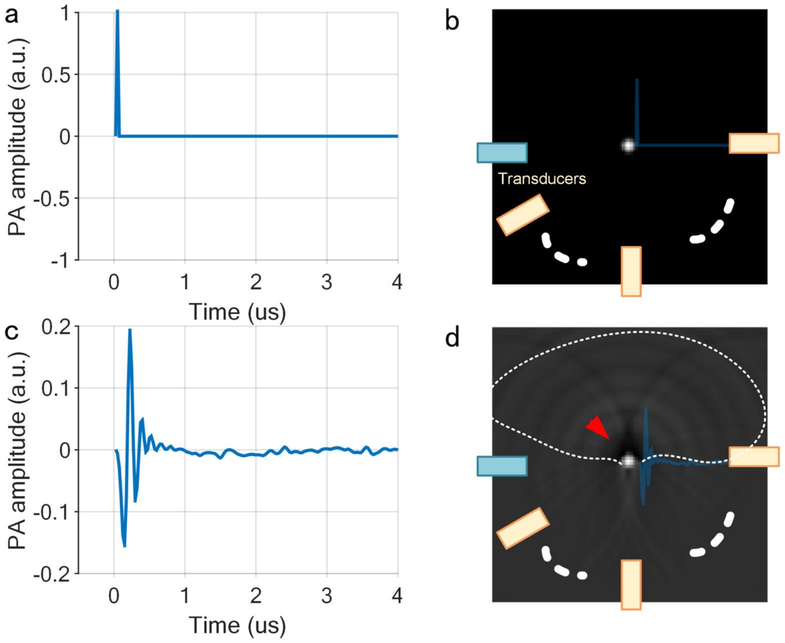

Supplementary Fig. 11 | Illustration on the artefact generation mechanism.

a, Response of ultrasound transducers of ideal (top) and nonideal (bottom) situation **b**, Corresponding reconstructed raw photoacoustic images. Consider the impulse response of an ultrasound transducer: ideally, the transducer has an infinite bandwidth, resulting in a single peak in the time domain that precisely matches the input pressure waveform (a, b). In practice, however, the response is bandlimited and may exhibit harmonically oscillating tails following the excitation (c). These tails are also projected into the image during signal reconstruction, as indicated by the blue transducer and its projection line. This leads to 'shadows' around the point source (highlighted by the red arrow) and 'ripples' (encircled by the white dashed line), which are recognized as artefacts in raw PA images (d). While these artefacts are weak in amplitude, they follow the same temporal decoding curve as the point source in PATTERN, leading to their erroneous interpretation as PATTERN signals.

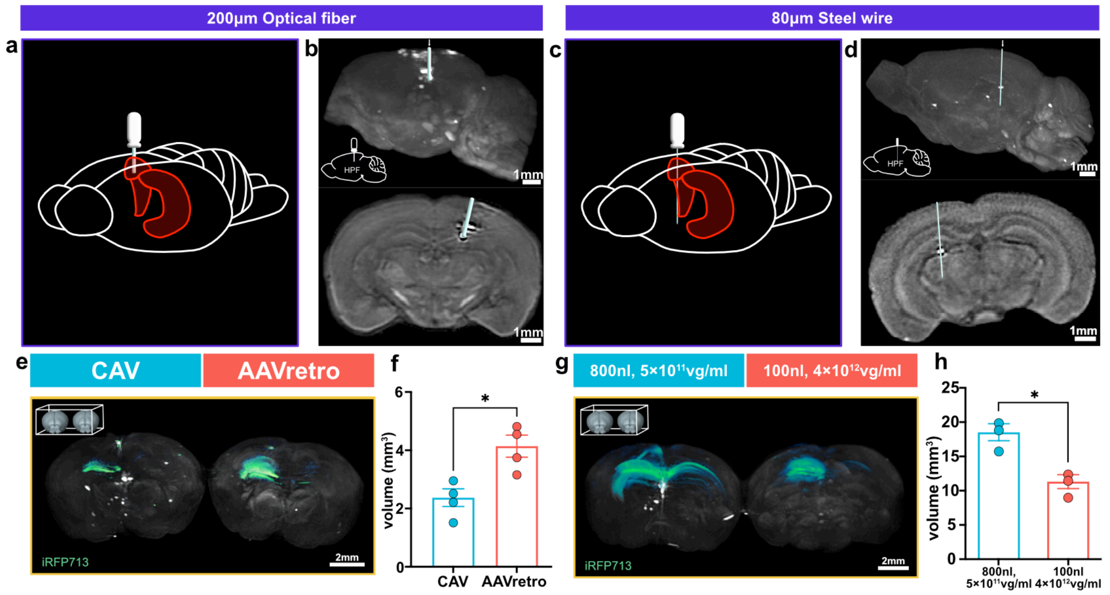

Supplementary Fig. 12 | PATTERN Imaging for visualizing implants in the brain and quantifying viral vector expression.

a, c, Implants diagrams of 200μm core diameter optical fiber, usually used in optogenetics and in-vivo fluorescence recording (a), and 80μm core diameter steel wire which was used to simulate the neuropixel (c). The experiment was repeated 3 times with similar results. **b, d**, 3D (top) and coronal (bottom) images via PATTERN of implants traces by using DIR. The DiR signals were unmixed using a linear unmixing method on raw PA images of three wavelengths (690 nm, 750 nm and 830 nm). Subsequently, based on the distribution pattern of DiR in the brain, the implants were modeled, and their positions and attitudes were calculated. Finally, these implants were rendered collectively with the brains to provide a complete visualization. **e, g**, Comparison of two different injection strategies, CAV (n = 4) vs. AAVretro (n = 4) (e) and different volume×titer (n = 3, n = 3) (g). Two brains were imaged by PATTERN in the same field. **f, h**, Statistical results of (e) and (g). Difference between means ± SEM are presented as: 1.769 ± 0.4847 (f), -7.203 ± 1.600 (h) T-test was used to test the difference. The significance levels were set to P =0.05. Significant for comparison: *p < 0.05; **p < 0.01; ***p < 0.001; ****p < 0.0001. Error bar length: 2 * mean squared error.

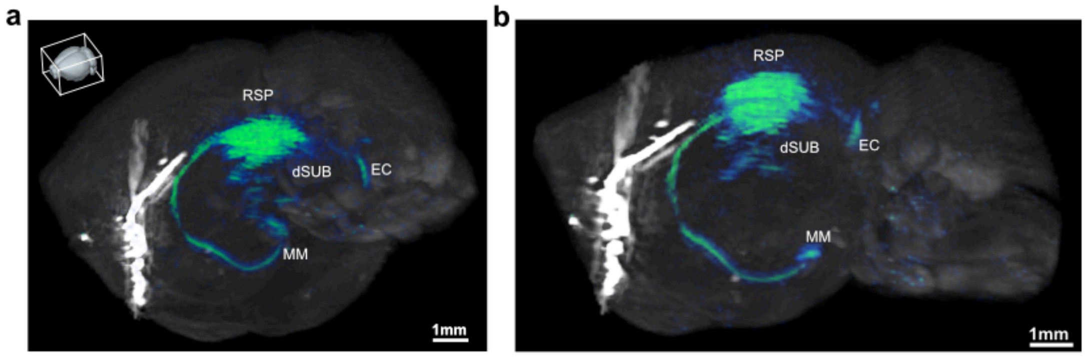

Supplementary Fig. 13 | Projections from dSub to RSP, MM and EC.

a, 3D-view image via PATTERN of the projection pattern of dSub. **b**, MIP imaging from the side of (a). The experiment was repeated 5 times with similar results.

a

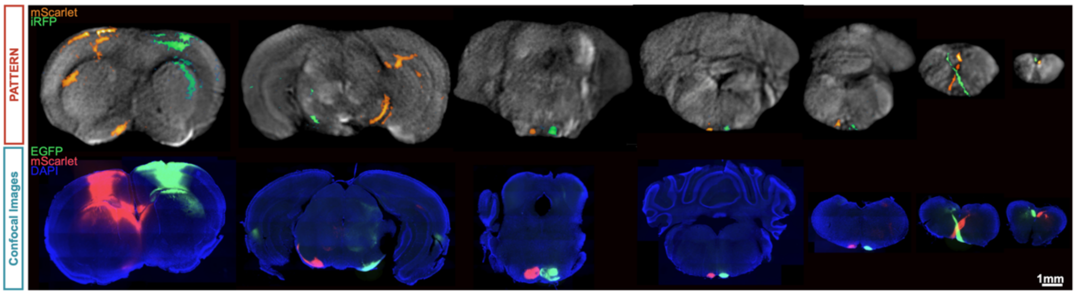

Supplementary Fig. 14 | Comparison of two imaging modalities of long-range projection.

a, Coronal sections images via PATTERN (top) and fluorescent microscopy (bottom) at the local and various projection sites. The experiment was repeated 3 times with similar results.

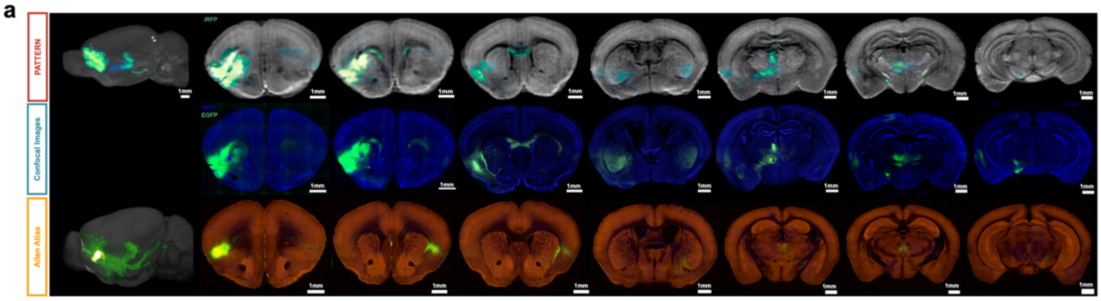

Supplementary Fig. 15 | Comparison of two imaging modalities of AI output.

a, Coronal section images via PATTERN (top), confocal microscopy (middle) and Allen Mouse Brain Connectivity Atlas, connectivity.brain-map.org/projection/experiment/262536037 (bottom) at the local and various projection sites. The experiment was repeated 5 times with similar results.

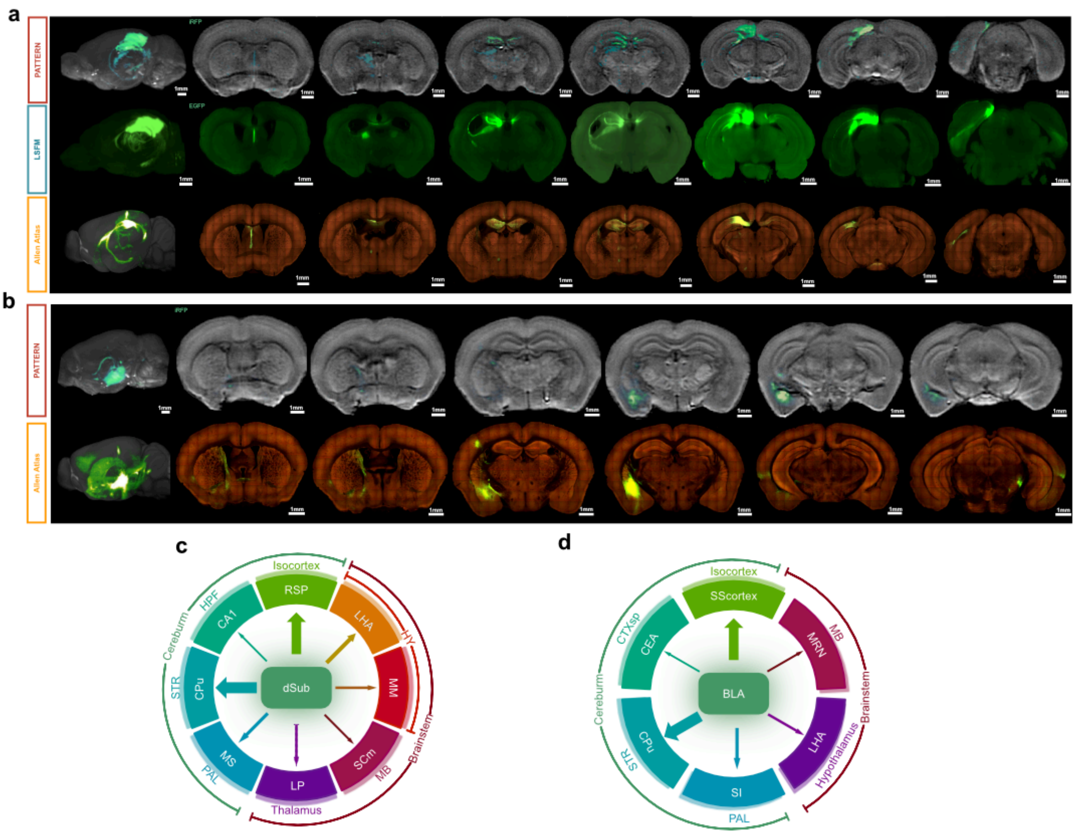

Supplementary Fig. 16 | Comparison of two imaging modalities of dSub and BLA output.

a, Coronal sections images via PATTERN (top), LSFM (middle) and Allen Mouse Brain Connectivity Atlas (bottom), connectivity.brain-map.org/projection/experiment/116900714. **b**, Coronal section images via PATTERN (top) and Allen Mouse Brain Connectivity Atlas (bottom), connectivity.brain-map.org/projection/experiment/120282354. The experiment was repeated 5 times with similar results. **c**, **d**, The major downstream diagram of dSub and BLA.

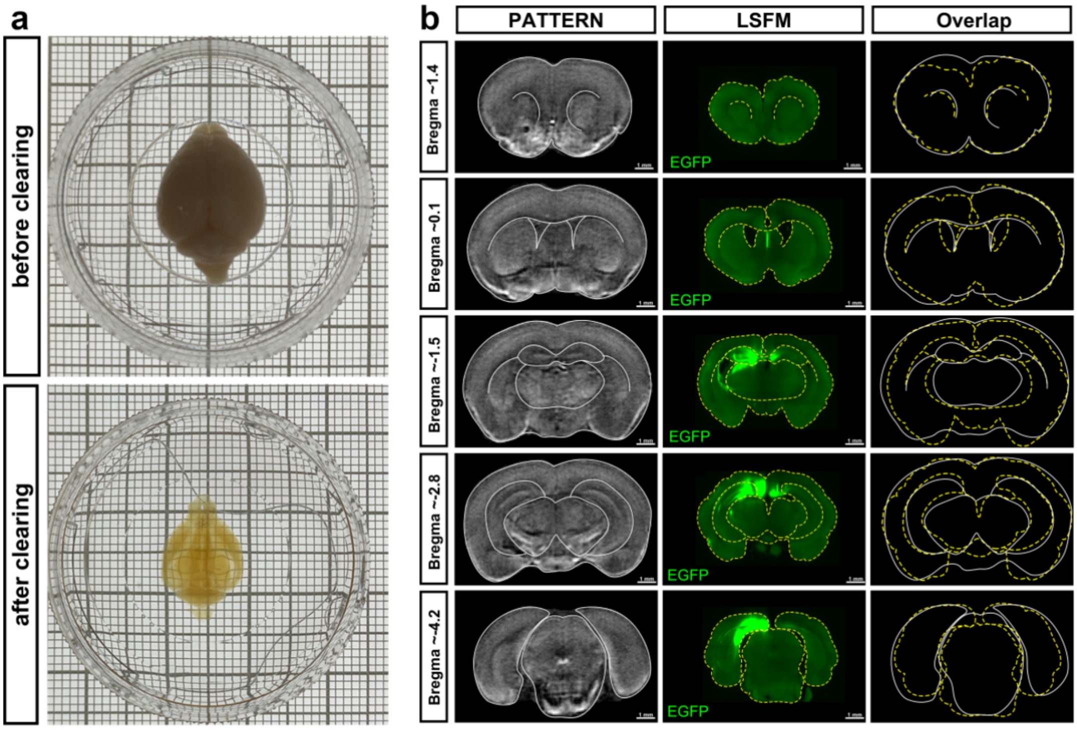

Supplementary Fig. 17 | The brain underwent anisotropic deformation after clearing with PEGASOS.

a, The physical diagram of the developmental malformed brain before and after clearing with PEGASOS. **b**, Brain shrinkage is anisotropic after clearing. The brain which injected EGFP-iRFP713 fusion virus into dSub was performed PATTERN before PEGASOS and LSFM. The borders of major structures of PATTERN and LSFM were highlighted in white solid or yellow broken lines. Left, representative coronal-section of PATTERN, iRFP713 signal is not shown. Middle, representative coronal-section of LSFM. Right, the border lines of LSFM were ratio-metricly enlarged to compare with the PATTERN lines and they showed overlap.

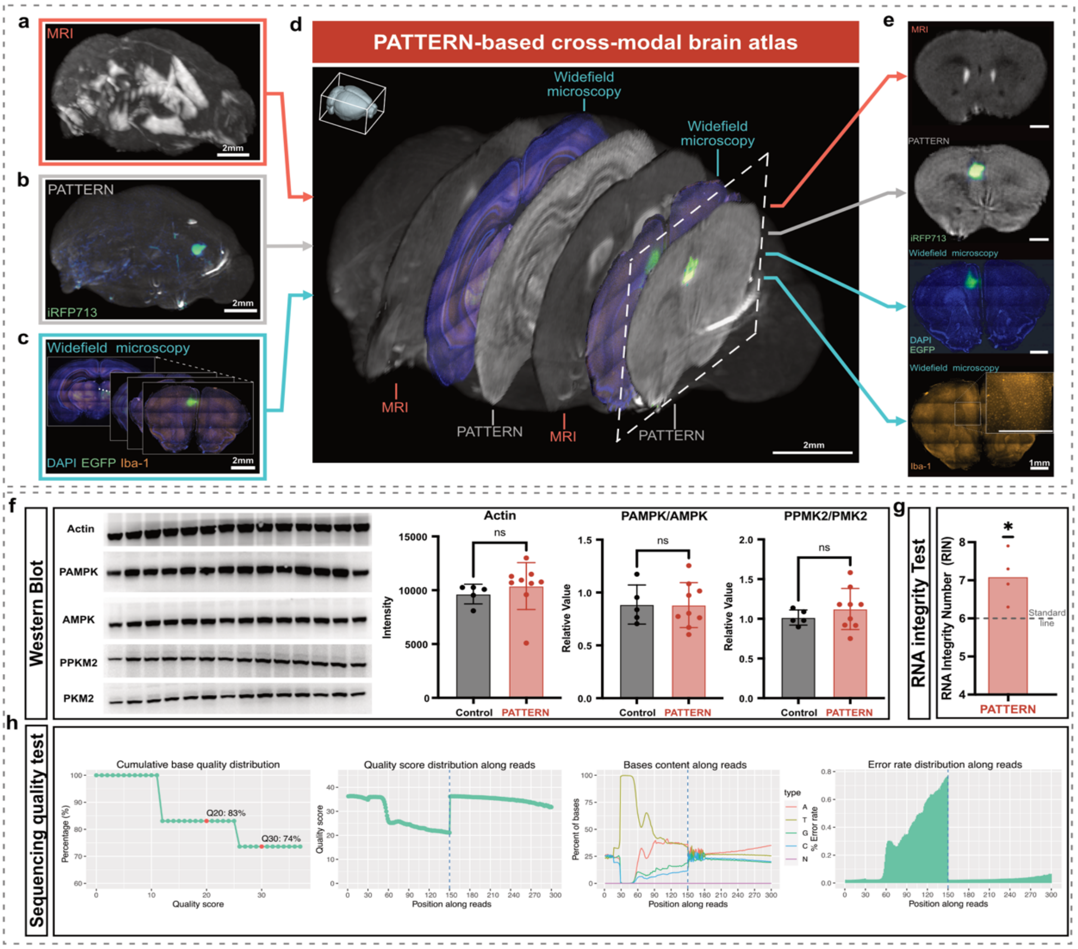

Supplementary Fig. 18 | Verification of PATTERN-based cross-modal analysis.

a-d, 3D images via MRI (a), PATTERN (b), sliced immunohistology (c) and PATTERN-based cross-modal brain atlas (d). **e**, Different modalities of the same coronal section. **f**, Protein content detection by western blot. Left column: western bolt images. Specifically, the samples of lane 1, 3, 5, 7, and 9 were control group without treatment while the others were imaged by PATTERN. Right columns: statistical results. n = 5 (Control), n = 9 (PATTERN). Difference between means ± SEM are presented as: 742.9 ± 1037 (f-actin), -0.005067 ± 0.1137 (f-PAMPK/AMPK), 0.1074 ± 0.1217 (f-PPMK2/PMK2), T-test was used to test the difference. The significance levels were set to P =0.05. Significant for comparison: *p < 0.05; **p < 0.01; ***p < 0.001; ****p < 0.0001. Error bar length: 2 * mean squared error. **g**, RNA integrity tests (agilent 2000) of 4 samples after PATTERN imaging. Data are presented as 7.1 (mean). One-sample t test was used to test the differenece, p-value = 0.047. Significant for comparison: *p < 0.05; **p < 0.01; ***p < 0.001; ****p < 0.0001. **h**, Quality test during RNA sequencing.

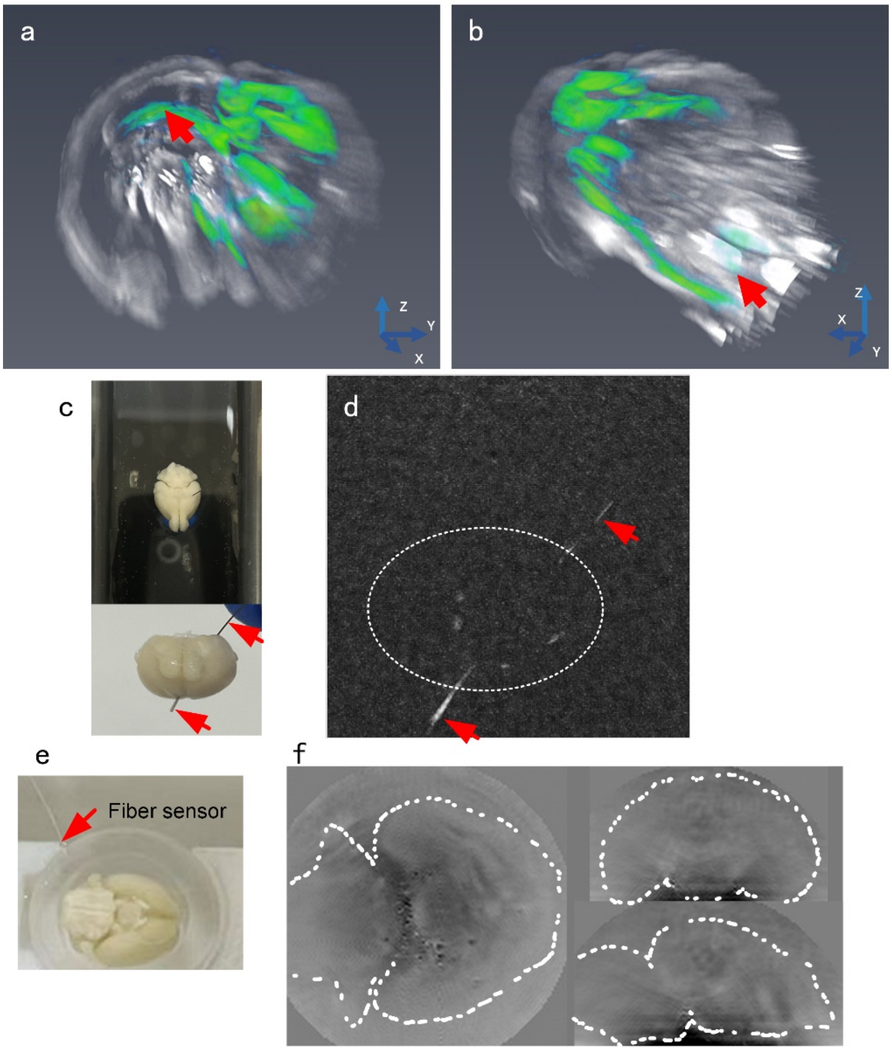

Supplementary Fig. 19 | Brain imaging using other systems.

a, b, Imaging results using a full ring array system[1]. **c**, Photos of the brain sample imaged by a hemispherical array system (Nexus 128, Endra). **d**, Imaging result of the hemispherical array system. Dashed white circle delineates the position of the brain sample. We illuminated the sample using a 700 nm laser and performed the angular scan in 120 steps with 30 averages per step, the entire scanning process took 3.9 minutes. It is important to acknowledge that the hemispherical array system was acquired in 2017 and has undergone extensive use. Consequently, its current performance may not accurately reflect that of a typical hemispherical array system. **e**, Brain sample inside the fiber sensor-based photoacoustic mesoscope. **f**, Imaging results of the fiber-optic mesoscope[2], the profile of the brain was delineated with white dashed lines.

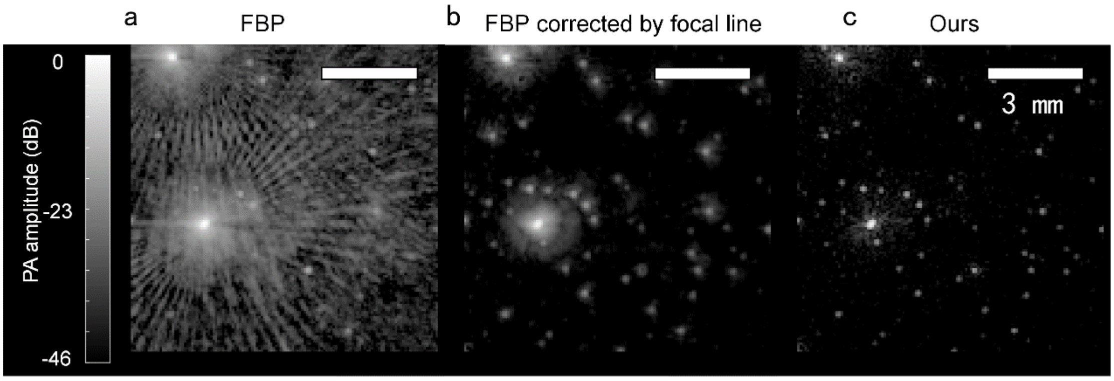

Supplementary Fig. 20 | Comparison with filtered back-projection reconstruction.

a, Reconstruction results using 3D filter back projection method (FBP). **b**, Reconstruction results using FBP with corrections on spatial impulse response (SIR)[3-5]. The corrections was made using the "focal-line" technology[4]. **c**, Reconstruction results using the purposed method in the current work.

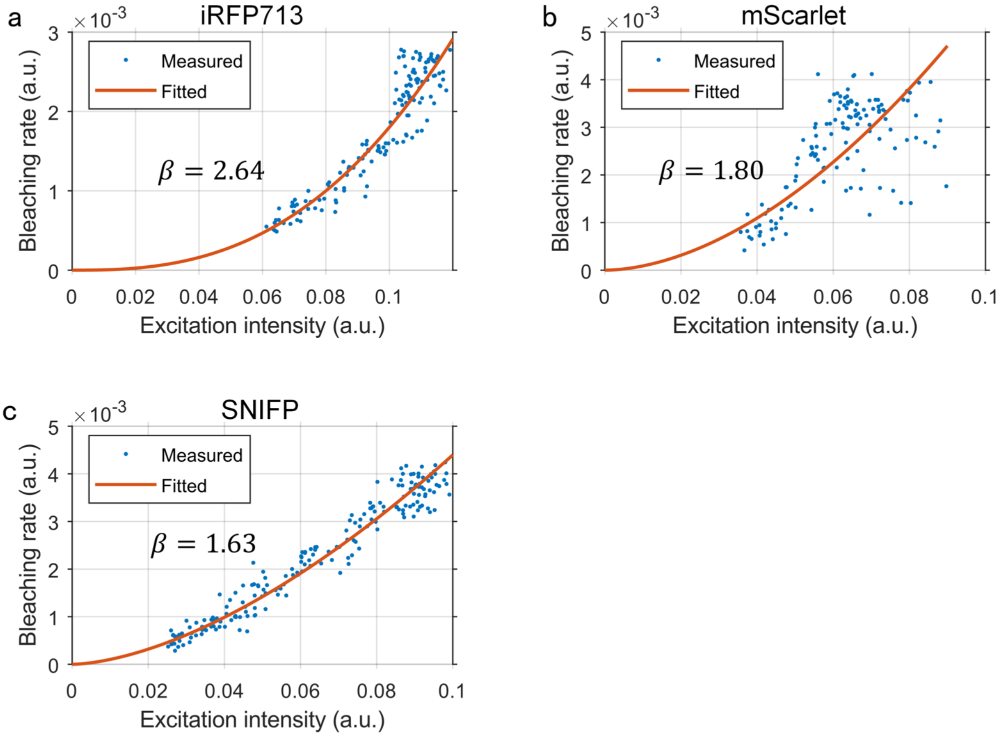

Supplementary Fig. 21 | The non-linearity of different fluorescent proteins.

a-c, Photobleaching rates of different proteins have different intensity power dependences. (a), iRFP713, (b), mScarlet, (c), SNIFP.

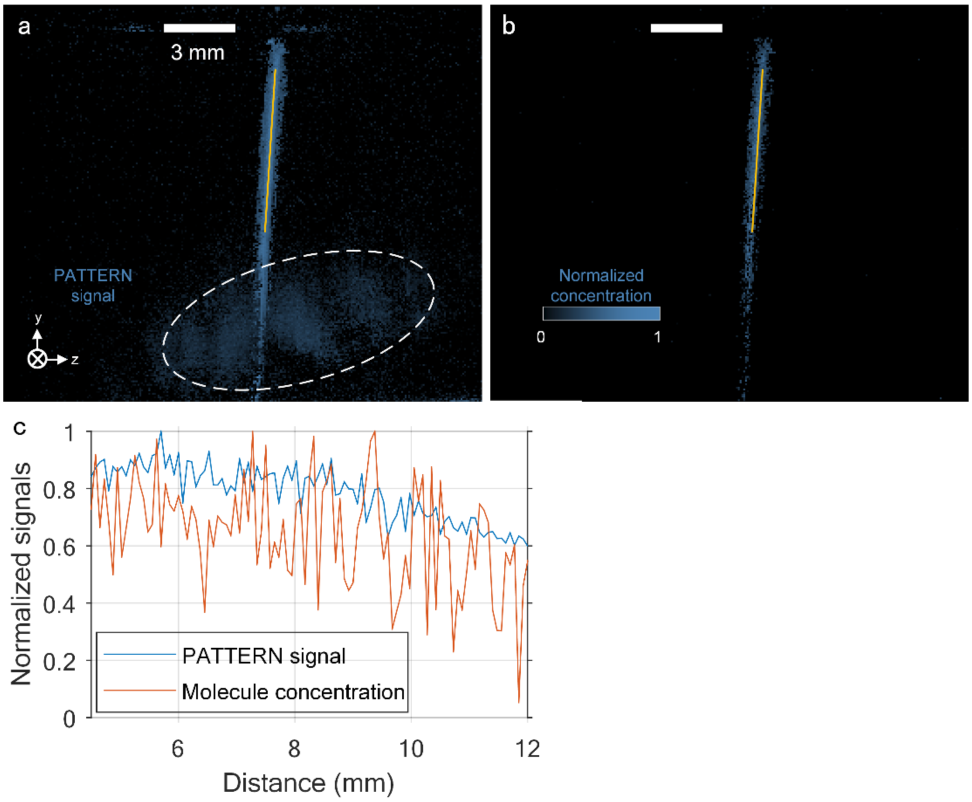

Supplementary Fig. 22 | Comparison of PATTERN signal and quantified PA signals.

a, PATTERN image of a tube filled with SNIFP. White dashed circle: artefacts or false positive PATTERN signals can be effectively reduced, as their bleaching rates are typically too rapid to align with their amplitudes. **b**, Molecule concentration image corresponding to (a) reconstructed using the non-linear-based quantification method. **c**, Profiles of the lines in (a) and (b). This method demands high precision in fitting since errors or noise are magnified during the additional calculation process. This requirement can hinder accurate quantification

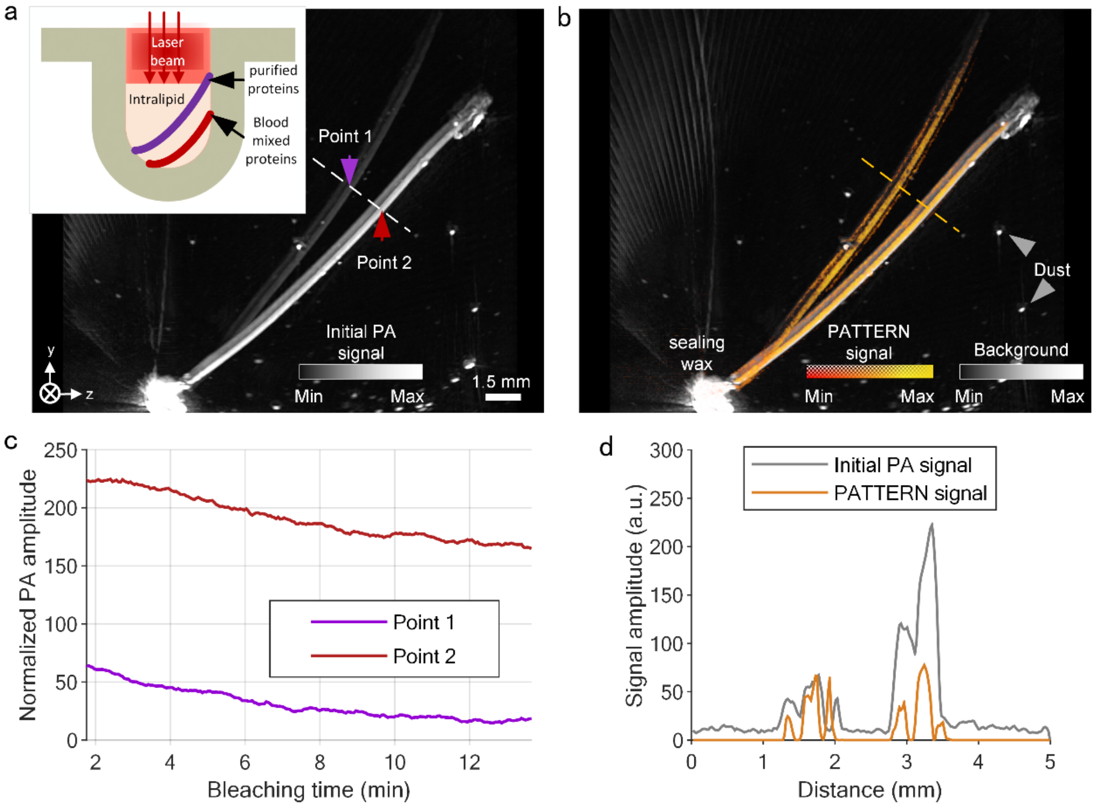

Supplementary Fig. 23 | Performance of PATTERN in the presence of blood.

a, Photoacoustic image depicting two tubes, one filled with purified iRFP713 (upper left) and the other with a mixture of iRFP713 and bovine blood (lower right). Inset: experimental schematic. **b**, PATTERN image corresponding to (a). **c**, Bleaching curves of the two points indicated by the arrows in (a). **d**. Profiles along the dashed lines in (a) and (b).

Supplementary Table

Supplementary Table 1 Performance comparison of different methods of whole-brain optical imaging.

Performance comparison of different methods of whole-brain optical imaging

Different Methods	Sample Preparation			Imaging			Data Analyzing
Typical method	Sample preparation (Tissue Clearing) method	Whether to fix the sample	Sample preparation time	Typical voxel size	FOV	Data collection time (single color)	Reconstruction time
PATTERN (Our system)	None (non-destructive)	Both fixed and fresh samples are available	0	75 μm×75 μm×75 μm	2 cm×2 cm	20.5 mins per mouse brain	8 h
STP[10]	Agarose-embedding, vibrating sectioning	Fixed	10-14 days	0.5 μm×0.5 μm×50 μm	1.66 mm×1.66 mm	24 h per mouse brain	(Not mention)
FAST[11]	Agarose--embedding, vibrating sectioning	Fixed	12-25 h	0.7 μm×0.7 μm× 5 μm	1.43 mm×1.43 mm	2.4-10 h per mouse brain	16-30 h
fMOST[12]	Resin- embedded sectioning	Fixed	5 days	0. 32 μm× 0. 32 μm×1 μm	0.65 mm×0.65 mm	111h per mouse brain	3-5 days
Different clearing methods combined with Zeiss LightsheetZ.1 (5× detection lens, zoom 0.36×) (Our data)	uDISCO[6]	Fixed	5 days	2.5 μm× 2.5 μm×6.5 μm	3.4 mm×3.4 mm	1.5 h	1 days
	CUBIC[7]	Fixed	7-21 days				
	PAGESOS[8]	Fixed	7 days				
	CLARITY[9]	Fixed	7 days				

	Type	PATTERN	Sectioning Tomography	Tissue Clearing + Light-sheet microscopy

Supplementary Methods

MATLAB code for Monte Carlo simulation

```
    load([datapath2,'simulate_label_brain.mat']); % load labels of brain and holder
    vol = uint8(simulate_label_brain);

    cfg.vol = vol;
    cfg.unitinmm=19.2/256; % set the grid size to be the same with the images
    cfg.prop=[0.002,1,1,1.37;                          % water
        0.002,20,0.98,1.37;   % agar
        0.005,20,0.9,1.37; % brain
        ];              % standard tissue

    cfg.nphoton=1e9;
    cfg.issrcfrom0=1;
    cfg.srcpos=[128 1 128];% center of the beam
    cfg.tstart=0;
    cfg.tend=5e-9;
    cfg.tstep=5e-9;

    % set light source
    cfg.srcdir=[0 1 0];
    cfg.srctype='disk';
    cfg.srcparam1=[106 0 0 0]; % beam radius of 7.95 mm
    cfg.isreflect=0;
    cfg.autopilot=1;
    cfg.gpuid=1;
    cfg.debuglevel='P';

    cfg.outputtype='energy';
    flux=mcxlab(cfg);
```

Supplementary References

1. Cui, M. *et al.* Adaptive photoacoustic computed tomography. *Photoacoustics* **21**, 100223 (2021).
2. Chen, Y. *et al.* Photoacoustic Mouse Brain Imaging Using an Optical Fabry-Pérot Interferometric Ultrasound Sensor. *Frontiers in neuroscience* **15**, 572 (2021).
3. Lu, T. *et al.* Full-frequency correction of spatial impulse response in back-projection scheme using space-variant filtering for optoacoustic mesoscopy. *Photoacoustics* **19**, 100193 (2020). https://doi.org:https://doi.org/10.1016/j.pacs.2020.100193
4. Xia, J. *et al.* Three-dimensional photoacoustic tomography based on the focal-line concept. *Journal of Biomedical Optics* **16**, 090505-090505-090503 (2011). https://doi.org:10.1117/1.3625576
5. Seeger, M. *et al.* Pushing the boundaries of optoacoustic microscopy by total impulse response characterization. *Nature Communications* **11**, 2910 (2020). https://doi.org:10.1038/s41467-020-16565-2
6. Pan, C. *et al.* Shrinkage-mediated imaging of entire organs and organisms using uDISCO. *Nature Methods* **13**, 859-867 (2016). https://doi.org:10.1038/nmeth.3964
7. Matsumoto, K. *et al.* Advanced CUBIC tissue clearing for whole-organ cell profiling. *Nature Protocols* **14**, 3506-3537 (2019). https://doi.org:10.1038/s41596-019-0240-9
8. Jing, D. *et al.* Tissue clearing of both hard and soft tissue organs with the PEGASOS method. *Cell Research* **28** (2018). https://doi.org:10.1038/s41422-018-0049-z
9. Tomer, R., Ye, L., Hsueh, B. & Deisseroth, K. Advanced CLARITY for rapid and high-resolution imaging of intact tissues. *Nature Protocols* **9**, 1682-1697 (2014). https://doi.org:10.1038/nprot.2014.123
10. Ragan, T. *et al.* Serial two-photon tomography for automated ex vivo mouse brain imaging. *Nature methods* **9**, 255-258 (2012). https://doi.org:10.1038/nmeth.1854
11. Seiriki, K. *et al.* High-Speed and Scalable Whole-Brain Imaging in Rodents and Primates. *Neuron* **94**, 1085-1100.e1086 (2017). https://doi.org:10.1016/j.neuron.2017.05.017
12. Zhong, Q. *et al.* High-definition imaging using line-illumination modulation microscopy. *Nature Methods* **18**, 309-315 (2021). https://doi.org:10.1038/s41592-021-01074-x
