# Supplementary material for: Photoacoustic Tomography with Temporal Encoding Reconstruction (PATTERN) for cross-modal individual analysis of the whole brain
Source: Nat Commun. 2024 May 18;15:4228. doi: 10.1038/s41467-024-48393-z (PMC11102525; doi:10.1038/s41467-024-48393-z)
Supplement: Supplementary file 3 — Description of Additional Supplementary Files [file 41467_2024_48393_MOESM3_ESM.pdf]

Description of Additional Supplementary Files

File name: Supplementary Movie 1
Description: The process of PATTERN imaging
The PATTERN system was upgraded from a conventional PACT system with a half-ring array transducer. To image in 3D, the PATTERN system scans linearly along the elevational direction and is rotated to vary the scan direction. The 3D images acquired at different scan angles are combined to reconstruct a 3D volume with near isotropic resolution. A typical bleaching process includes 8 Translation-rotational scan cycles. Signals and image features are derived from temporal encoding as illustrated using pseudocolor superimposed on the grayscale background.

File name: Supplementary Movie 2
Description: Visualizing the whole brains of different species via PATTERN system
Whole-brain images of various animals, including mouse (first column), rat (second column), ferret (third column), and marmoset (forth column), are acquired via PA imaging.. The top row shows the three-dimensional rendering, while the bottom row displays coronal planes scanned from anterior to posterior. The green dots visible in the brains of the mouse, rat, and ferret represent injected DIR dye.

File name: Supplementary Movie 3
Description: Visualizing the projections from cortex to spinal cord of the whole CNS via PATTERN
Left: 3D PATTERN imaging of the dual-color whole CNS for bilateral motor cortex projections (iRFP713: green and mScarlet: orange). Right: 3D PATTERN imaging of unilateral motor cortex projections (iRFP713: orange).

File name: Supplementary Movie 4
Description: PATTERN for isotropically visualizing neural connectivity of the brain
First video: the brain-wide projection map of a mouse anterior insular cortex (iRFP713; green): coronal (upper left), sagittal (upper right), transverse (lower left) and 3D structure (lower right). Second video: the brain-wide projection map of a rat hippocampus (iRFP713: green): coronal (upper left), sagittal (upper right), transverse (lower left) and 3D structure (lower right).

File name: Supplementary Movie 5
Description: Three-dimensional PATTERN and LSFM images of the same mouse brain
A mouse brain with AAV injection in dorsal subiculum(dSub) went through PATTERN imaging before tissue clearing and LSFM imaging. Left: data acquired by LSFM, green signal shows the projection from dSub. Middle: data acquired by PATTERN, orange signal shows the projection from dSub. Right: Merged display of the LSFM and PATTERN images.

File name: Supplementary Movie 6
Description: Cross-modal correction of fMOST and LSFM images via PATTERN system
Two brain samples went through PA imaging before fMOST and LSFM imaging, the self-to-self template acquired by PA imaging was used to correct the anisotropic morphological changes. Top: brain sample processed by PA imaging and fMOST, red signal shows the morphology. Bottom: brain sample processed by PA imaging and LSFM combined with tissue clearing, green signal shows the morphology. Left: the original deformed image acquired by fMOST and LSFM. Middle: the self-to-self template acquired by PA imaging. Right: fMOST and LSFM images after PA-based cross-modal correction.

File name: Supplementary Movie 7
Description: Cross-modal correction performed on an AD rat brain and a malformed brain
Two brain samples went through PA imaging before tissue clearing and LSFM imaging, the self-to-self template acquired by PA imaging was used to correct the anisotropic morphological changes. Top: an aged rat brain with Alzheimer’s disease processed by PA imaging and iDISCO, red signal shows the Aβ40/42 and morphology. Bottom: a malformed mouse brain with hydrocephalus processed by PA imaging and PEGASOS, green signal shows the morphology. Left: the original deformed data acquired by LSFM. Middle: the self-to-self template acquired by PA imaging. Right: LSFM data after PA-based cross-modal correction.
