# Supplementary material for: Photoacoustic Tomography with Temporal Encoding Reconstruction (PATTERN) for cross-modal individual analysis of the whole brain
Source: Nat Commun. 2024 May 18;15:4228. doi: 10.1038/s41467-024-48393-z (PMC11102525; doi:10.1038/s41467-024-48393-z)
Supplement: Supplementary file 11 — Reporting Summary [file 41467_2024_48393_MOESM11_ESM.pdf]

nature portfolio

Corresponding author(s): Cheng Ma; Bo Lei

Last updated by author(s): Mar 26, 2024

Reporting Summary

Nature Portfolio wishes to improve the reproducibility of the work that we publish. This form provides structure for consistency and transparency in reporting. For further information on Nature Portfolio policies, see our Editorial Policies and the Editorial Policy Checklist.

Statistics

For all statistical analyses, confirm that the following items are present in the figure legend, table legend, main text, or Methods section.

n/a	Confirmed	
☐	☒	The exact sample size (*n*) for each experimental group/condition, given as a discrete number and unit of measurement
☐	☒	A statement on whether measurements were taken from distinct samples or whether the same sample was measured repeatedly
☐	☒	The statistical test(s) used AND whether they are one- or two-sided<br>*Only common tests should be described solely by name; describe more complex techniques in the Methods section.*
☒	☐	A description of all covariates tested
☐	☒	A description of any assumptions or corrections, such as tests of normality and adjustment for multiple comparisons
☐	☒	A full description of the statistical parameters including central tendency (e.g. means) or other basic estimates (e.g. regression coefficient) AND variation (e.g. standard deviation) or associated estimates of uncertainty (e.g. confidence intervals)
☐	☒	For null hypothesis testing, the test statistic (e.g. *F*, *t*, *r*) with confidence intervals, effect sizes, degrees of freedom and *P* value noted<br>*Give P values as exact values whenever suitable.*
☒	☐	For Bayesian analysis, information on the choice of priors and Markov chain Monte Carlo settings
☒	☐	For hierarchical and complex designs, identification of the appropriate level for tests and full reporting of outcomes
☒	☐	Estimates of effect sizes (e.g. Cohen's *d*, Pearson's *r*), indicating how they were calculated

Our web collection on statistics for biologists contains articles on many of the points above.

Software and code

Policy information about availability of computer code

Data collection	Photoacoustic signal data was collected using MarsonicsDAQ128 with its software.
Data analysis	Photoacoustic image reconstruction and related temporal unmixing process was carried out by custom codes of Python 3.8. Artefacts removal was achieved by using a customized denoising convolutional neural network (DnCNN). Registration of images was performed by ANTS (Advanced Normalization Tools). Additional data analyses were performed using MATLAB R2022a, Amira2019 and imageJ 1.53t. Image rendering for visualization was managed in Amira2019. Imaging data of confocal microscopy and axio-scan Z1 was processed in Zen3.6. Other imaging data was processed in Imaris9.72 or Amira2019. Sequencing data was processed by customized code based on Seurat.

For manuscripts utilizing custom algorithms or software that are central to the research but not yet described in published literature, software must be made available to editors and reviewers. We strongly encourage code deposition in a community repository (e.g. GitHub). See the Nature Portfolio guidelines for submitting code & software for further information.

Data

Policy information about availability of data

All manuscripts must include a data availability statement. This statement should provide the following information, where applicable:

- Accession codes, unique identifiers, or web links for publicly available datasets
- A description of any restrictions on data availability
- For clinical datasets or third party data, please ensure that the statement adheres to our policy

Raw data of spatial transcriptome have been deposited in the National Center for Biotechnology Information's Sequence Read Archive with accession numbers PRJNA1091401. The processed spatial transcriptome analysis is available at https://github.com/CaA2318777/PATTERN/tree/main/SpatialTranscriptome. Raw data supporting the findings of the present study are provided as online materials for this paper . All other data that supports this study are available from the corresponding author upon request due to their large file size. Processed photoacoustic imaging data generated in this study have been deposited in the figshare database under accession link https://figshare.com/s/c0f6139f729b97b21028 . Source data are provided as a Source Data file. We have uploaded all the data and code produced in this project to a public database. The link to access them is: https://github.com/CaA2318777/PATTERN.

Research involving human participants, their data, or biological material

Policy information about studies with human participants or human data. See also policy information about sex, gender (identity/presentation), and sexual orientation and race, ethnicity and racism.

Reporting on sex and gender	not applicable
Reporting on race, ethnicity, or other socially relevant groupings	not applicable
Population characteristics	not applicable
Recruitment	not applicable
Ethics oversight	not applicable

Note that full information on the approval of the study protocol must also be provided in the manuscript.

Field-specific reporting

Please select the one below that is the best fit for your research. If you are not sure, read the appropriate sections before making your selection.

☒ Life sciences ☐ Behavioural & social sciences ☐ Ecological, evolutionary & environmental sciences

For a reference copy of the document with all sections, see nature.com/documents/nr-reporting-summary-flat.pdf

Life sciences study design

All studies must disclose on these points even when the disclosure is negative.

Sample size	Sample size was described in each Figure legend. Overall, we used at least three samples for each statistical experiment to demonstrate replicability while keeping the number of experimental animals to a minimum. Except for PATTERN imaging of the marmoset brain, because our primary goal was to demonstrate the feasibility of imaging the marmoset brain without purposefully quantitative analysis, which we did not repeat in view of minimizing the overuse of non-human primate samples and the difficulty of obtaining such samples. We did not use any statistical method to predetermine sample size, but our sample sizes are similar to those reported in previous publications.
Data exclusions	We did not include several mice and ferrets that were part of early attempts to use PATTERN. These animals were excluded due to poor viral expression and human error.
Replication	For each experiment, we did the corresponding pre-experiments or repeated experiments. For each experiment, we repeated it at least three times and obtained consistent results, and finally selected one of them as the presentation data
Randomization	For experiments involving experimental and control groups, animals were randomly divided into control and experimental groups
Blinding	We have different people doing imaging and biological experiments. The students in charge of imaging did not know the biological grouping, and the students in charge of biological experiment did not know the imaging results in advance when analyzing the data unless it is necessary.

Reporting for specific materials, systems and methods

We require information from authors about some types of materials, experimental systems and methods used in many studies. Here, indicate whether each material, system or method listed is relevant to your study. If you are not sure if a list item applies to your research, read the appropriate section before selecting a response.

Materials & experimental systems

n/a	Involved in the study	
☐	☒	Antibodies
☒	☐	Eukaryotic cell lines
☒	☐	Palaeontology and archaeology
☐	☒	Animals and other organisms
☒	☐	Clinical data
☒	☐	Dual use research of concern
☒	☐	Plants

Methods

n/a	Involved in the study	
☒	☐	ChIP-seq
☒	☐	Flow cytometry
☐	☒	MRI-based neuroimaging

Antibodies

Antibodies used

mouse anti-β-actin, Biodee, DE0620, RRID:AB_2737288, 1:8000; rabbit anti-AMPK, Cell Signaling Technology, CST2532, RRID:AB_330331, 1:1000; rabbit anti-PAMPK, Cell Signaling Technology, CST2535, RRID:AB_331250, 1:1000; rabbit anti-PKM2, Cell Signaling Technology, CST3198, RRID:AB_2252325, 1:1000; rabbit anti-PPKM2, Cell Signaling Technology, CST3827, RRID:AB_1950369, 1:1000; horse Anti-mouse IgG, HRP-linked Antibody, Cell Signaling Technology, 7076s, RRID:AB_330924, 1:2000; goat Anti-rabbit IgG, HRP-linked Antibody, Cell Signaling Technology, 7074s, RRID:AB_2099233, 1:2000; rabbit anti-Iba-1, Cell Signaling Technology, 17198, RRID:AB_2820254, 1:1000

Validation

https://www.cellsignal.com/products/primary-antibodies/iba1-aif-1-e4o4w-xp-rabbit-mab/17198?_=1681874381075&Ntt=17198&tahead=true
http://www.biodee.net/Show/index/cid/95/id/1398.html
https://www.cellsignal.cn/products/primary-antibodies/ampka-antibody/2532?_=1681875319938&Ntt=2532&tahead=true
https://www.cellsignal.cn/products/primary-antibodies/phospho-ampka-thr172-40h9-rabbit-mab/2535?_=1681875355959&Ntt=2535&tahead=true
https://www.cellsignal.cn/products/primary-antibodies/pkm2-antibody/3198?site-search-type=Products&N=4294956287&Ntt=3198&fromPage=plp&_requestid=1466263
https://www.cellsignal.cn/products/primary-antibodies/phospho-pkm2-tyr105-antibody/3827?site-search-type=Products&N=4294956287&Ntt=3827&fromPage=plp&_requestid=1468085
https://www.cellsignal.cn/products/secondary-antibodies/anti-mouse-igg-hrp-linked-antibody/7076?_=1681875770615&Ntt=7076&tahead=true
https://www.cellsignal.cn/products/secondary-antibodies/anti-rabbit-igg-hrp-linked-antibody/7074?site-search-type=Products&N=4294956287&Ntt=7074&fromPage=plp&_requestid=1475466

Animals and other research organisms

Policy information about studies involving animals; ARRIVE guidelines recommended for reporting animal research, and Sex and Gender in Research

Laboratory animals

Male and female C57BL/6 mice (8–16 postnatal weeks, 25-30 grams of weight), male and female BALB/c mice (8–16 postnatal weeks, 25-30 grams of weight), male and female wild-type SD (Sprague Dawley, 12-20 postnatal weeks) rats and AppNL-G-F rats, male and female ferrets and a female marmoset brain were used in these experiments.
All experimental procedures of rodents and ferrets were approved by the Institutional Animal Care and Use Committee (IACUC) of Tsinghua University and were performed using the principles outlined in the Guide for the Care and Use of Laboratory Animals of Tsinghua University.
The female marmoset brain sample we used was from Gao Lixia's lab in Zhejiang University. It was housed, maintained, and bred at the Zhejiang University Interdisciplinary Institute of Neuroscience and Technology (ZIINT) Non-Human Primate Center located at the Huajiachi Campus, Hangzhou, Zhejiang Province, China. All experimental procedures were approved by the Zhejiang University Animal Care and Use Committee.

Wild animals

We did not use any wild animal.

Reporting on sex

Except for one female marmoset, both sexes were used in the system construction, testing, and application experiments

Field-collected samples

We did not use any field-collected sample

Ethics oversight

All experimental procedures were approved by Institutional Animal Care and Use Committee (IACUC) of Tsinghua University, and were performed using the principles outlined in the Guide for the Care and Use of Laboratory Animals of Tsinghua University.

Note that full information on the approval of the study protocol must also be provided in the manuscript.

Magnetic resonance imaging

Experimental design

Design type
Resting-state or anesthetic-state

Design specifications
For each mouse we conducted one imaging session. During the acquisition, we collected structural data in less than 25 to 45 minutes after anesthetic. The total experiment time was approximately 1 hour.

Behavioral performance measures
Behavioral performance was not performed in this study.

Acquisition

Imaging type(s)
Structural

Field strength
9.4T

Sequence & imaging parameters
The mice were scanned in horizontal MRI scanners (9.4 T/30 cm, Bruker BioSpec 94/30, Germany, software ParaVision for MRI acquisition). Anesthesia was induced with 3% isoflurane (R5835, RWD Life Science) and maintained during scanning using 1.5% isoflurane supplemented with 93% oxygen. The body temperature was kept at 37? through the circulating water tank (SC100-S5P, THERMO HAAKE, USA), and the respiratory status is monitored in real time through the ERT module (Model 1030, SA Instruments Inc., USA). A T2-weighted structural image was acquired by using T2 _Turbo _ RARE sequecne with following parameters: Number of slices=59, TR = 5849 ms, TE = 33.79 ms, flip angle = 90°, FOV = 16 × 15 mm, matrix size = 212 × 212, slice thickness = 0.3 mm ETL=10, NEX=5, TA= 10min14sec. Total scan time including animal positioning was around 1 hours.

Area of acquisition
We collected data from the whole mouse brain.

Diffusion MRI ☐ Used ☒ Not used

Preprocessing

Preprocessing software
MRI data were preprocessed using Amira2019 to segment the brain region.

Normalization
No normalization was applied.

Normalization template
No normalization template was needed. All data analysis was conducted in the subject's individual space.

Noise and artifact removal
During data acquisition, the respiratory status of the mouse is monitored in real time through the ERT module (Model 1030, SA Instruments Inc., USA). The structural data was only collected between two respiratory period to avoid the locomotion.

Volume censoring
No volume censoring was applied.

Statistical modeling & inference

Model type and settings
No statistical model was used.

Effect(s) tested
The structure data was collected from anesthetic mouse. No task or stimulus was performed.

Specify type of analysis: ☒ Whole brain ☐ ROI-based ☐ Both

Statistic type for inference
(See Eklund et al. 2016)
No statistical analysis was performed.

Correction
No correction was performed.

Models & analysis

n/a	Involved in the study	
☒	☐	Functional and/or effective connectivity
☒	☐	Graph analysis
☒	☐	Multivariate modeling or predictive analysis
